# Two Sylvatic Rabies Re-Emergences in Central-Eastern Europe over the 2021–2022 Period: An Unprecedented Situation in Recent Years

**DOI:** 10.1155/2023/5589201

**Published:** 2023-11-10

**Authors:** Emmanuelle Robardet, Marcin Smreczak, Anna Orlowska, Peter Malik, Alexandra Nándori, Zuzana Dirbáková, Slavomír Jerg, Oleksii Rudoi, Ivan Polupan, Oxana Groza, Serghei Arseniev, Florica Barbuceanu, Vlad Vuta, Evelyne Picard-Meyer

**Affiliations:** ^1^Anses, Nancy Laboratory for Rabies and Wildlife, EURL for Rabies, Bâtiment H, Technopôle Agricole et Vétérinaire, CS 40 009, 54220, Malzéville Cedex, France; ^2^National Veterinary Research Institute, NRL for Rabies, 57 Partyzantow Avenue, Pulawy 24-100, Poland; ^3^National Food Chain Safety Office VDD, NRL for Rabies, Tabornok u 2, Budapest 1143, Hungary; ^4^State Veterinary and Food Institute, Veterinary Institute Zvolen, Pod Drahami 960 86, 918, Zvolen, Slovakia; ^5^The State Scientific and Research Institute of Laboratory Diagnostics and Veterinary Sanitary Expertise, 30 Donetska Street, Kyiv 03151, Ukraine; ^6^Republican Center of Veterinary Diagnostic, 3 Street Murelor Chisinau, Chisinau, Moldova; ^7^Faculty of Veterinary Medicine, Splaiul Independentei, 105, Bucharest, Romania; ^8^Institute for Diagnosis and Animal Health, NRL for Rabies and WOAH Reference Laboratory for Rabies, Dr Staicovici No. 63, Sector 5, Bucharest, Romania

## Abstract

The implementations of coordinated, standardised, and sustained oral rabies vaccination (ORV) campaigns over large areas have led to the almost elimination of sylvatic rabies from the European Union (EU) territory at the approach of 2020. The annual number of rabies cases reported within EU territory indeed dropped from around 13,000 cases in 1990 to less than 10 cases over the 2017–2019 period. Unfortunately, since 2020, the EU territory has faced two major rabies re-emergence events of rabies lyssavirus in non-flying animals. The first sylvatic rabies outbreak, already described, occurred in 2021 and 2022 in Poland in the Mazowieckie voivodeship, involving the Central Europe variant, while the second one affecting Romania, Hungaria, and Slovakia, increased considerably at the end of 2022 and involved the North East Europe variant only. Thus, Hungary and Slovakia that did not record a single case since 5–7 years, respectively, faced new rabies outbreaks in 2022. This article, therefore, presents these two epidemiological events and discusses the importance and challenges of maintaining ORV programmes and immune belts in the long term, particularly in a complicated context of a pandemic affecting organisation of human societies and of geopolitical conflicts.

## 1. Introduction

While rabies has been known as a deadly disease since antiquity, contemporary modelling estimates this infection is causing annually around 60,000 human deaths worldwide [1]. Rabies, which is 100% lethal as soon as clinical signs appear, indeed affects continuously and silently, mainly developing countries, particularly in African and Asian continents, which allows it to be qualified as a neglected disease despite being entirely preventable through vaccination and prompt post-exposure treatment [2]. While all the Lyssavirus genus species are potential causative agents for the rabies disease, the classical rabies lyssavirus (RABV) is largely predominant, with up to 99% of human cases due to domestic dog transmission [3].

**Figure 1:**
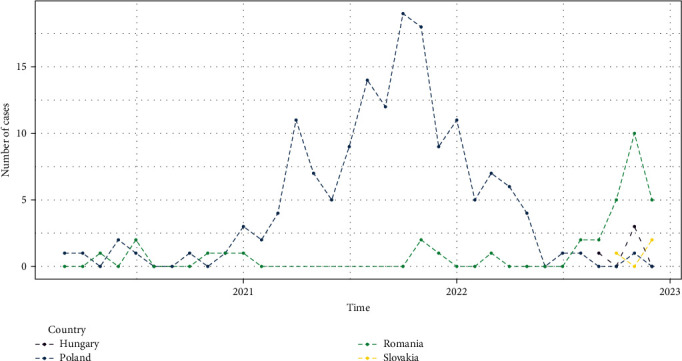
Monthly evolution of rabies cases reported in the EU from January 2020 to December 2022 (data source: Animal Disease Information System (ADIS) (europa.eu)).

**Figure 2:**
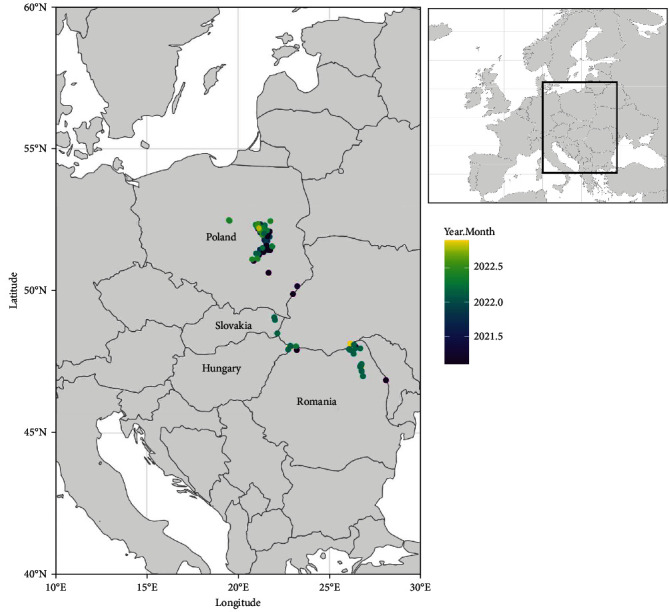
Spatial and temporal distribution of rabies cases detected in the EU during 2021–2022 (data source: Animal Disease Information System (ADIS) (europa.eu)).

In a large part of Europe, dog-mediated rabies has been eliminated in the past thanks to sanitary and dog control measures [4]. However, in 1935, a new rabies form, induced by the RABV virus and affecting principally red foxes (*Vulpes vulpes*), emerged close to the south of Kaliningrad and the Russo-Polish border [5, 6]. During the World War II, the disease spread centrifugally with a speed of 25–60 km/year, so that most of northern Europe was affected. The maximum geographical expansion of this epidemic was reached in the 1970s [7]. This sylvatic rabies was then controlled using new and unique technology of oral rabies vaccination (ORV) campaigns targeting red foxes [8, 9]. While the first and local attempt in 1978 in Switzerland were conclusive, the implementation of systematic, coordinated, sustained ORV campaigns in large areas was made possible thanks to an European-level organisation and a co-financing system [10, 11] leading to even considering an elimination of the disease by 2020 [12]. The maximum number of animal rabies cases reported within European Union (EU) territory indeed reached around 13,000 cases in 1990 (source: RBE data Queries | Rabies-Bulletin-Europe (who-rabies-bulletin.org)) while this figure constantly dropped to reach 7 cases in 2017, 7 in 2018, 5 in 2019 and 17 in 2020 (source: ADIS data Animal Disease Information System (ADIS) (europa.eu)). Unfortunately, the history will show a different scenario as the number of rabies cases strongly increased in 2021 in Poland and in 2022 in Romania (Figures 1 and 2). Also, Hungary and Slovakia, that were free since years, faced new animal rabies cases emergence in 2022. This manuscript attempts to present and discuss on these re-emergences that occurred over the period 2021–2022.

## 2. Rabies Surveillance and Phylogenetic Analysis

Rabies surveillance within the EU is based on a regulated framework and network surveillance involving all geographical administrative levels (local/regional/national/EU level). Animals showing abnormal behaviour suggestive of rabies, but also animals found dead, have to be considered as indicator animals and have to be tested in priority [2, 13]. Laboratory diagnosis of suspect animals is performed in EU countries based on WOAH reference techniques [14]. Fluorescent Antibody Test with positive cases confirmed by RT-CIT and/or RT-PCR (conventional or real-time) was performed in countries concerned by this study. Positive RABV samples detected within the EU over 2021–2022 (Poland: 11 from 2021 and 31 from 2022; Romania: 2 from 2021 and 27 from 2022; Hungary: 4 in 2022; Slovakia: 3 in 2022, considering the last positive case was found in 2022 and confirmed in January 2023) were submitted to phylogenetic analysis. Samples from bordering countries (1 sample from 2020, 9 samples from 2021 from Ukraine, 5 samples from 2021, and 11 from 2022 from the Republic of Moldova) as well as samples from Romania collected before the 2021–2022 period (2 from 2012, 2 from 2014, 2 from 2015, and 3 from 2020) were additionally included in the analysis for their proximity to the targeted areas. Nine samples were finally not exploitable (5 from Romania and 4 from Ukraine), leading to a total of 105 samples for RNA analysis (see supplementary material for details). Viral RNA was extracted from positive original samples for full-length *N* gene (1,353 bp) amplification. As a first step, an RT-PCR in one step was performed in a final volume of 15 *µ*L with 5 *µ*L of pure RNA extract, 3 *µ*L of Qiagen One-step RT-PCR kit (Qiagen, Les Ulys, France), 0.5 *µ*L of forward primer JW12 at 20 *µ*M (5′ ATG TAA CAC CYC TAC AATG-3′), 0.5 *µ*L of reverse primer PVN8-GT1 at 20 *µ*M (5′ AGT YTC TTC RGC CAT CTC-3′), 0.6 *µ*L dNTP (10 mM), 0.5 *µ*L RNAse inhibitor and 0.6 *µ*L Qiagen RT-PCR enzyme. Thermocycling conditions were one cycle of reverse-transcription for 30 min at 50°C, one cycle of denaturation for 15 min at 95°C, followed by 16 cycles of 30 s at 94°C, a 0.4°C touch-down decrease of the annealing temperature from 57°C to 51°C for 30 s and 2 min at 72°C, then 29 cycles for 30 s at 94°C, 30 s at 55°C, 2 min at 72°C, and a final extension for 10 min at 72°C. A second round of PCR was used to increase the detection threshold. About 2 *µ*L of pure RT-PCR products were amplified in a final volume of 20 *µ*L with 0.25 *µ*L of primers M13-JW12 20 *µ*M (5′-GTA AAA CGA CGG CCA GA TGT AAC ACC YCT ACA ATG-3′) and M13RevPVN8Bis 20 *µ*M (5′-CAG GAA ACA GCT ATG ACC TTG CTC ATA YTT GGG), 1 *µ*L of dNTP (2 mM), 1 *µ*L of MgCl_2_ (50 mM) and 0.25 *µ*L of Platinum Taq DNA polymerase (5 U/*µ*L) (Invitrogen). The PCR conditions were one cycle of denaturation for 2 min at 94°C, followed by 45 cycles for 30 s at 94°C, 30 s at 48°C and 2 min at 72°C and a final extension for 7 min at 72°C. Finally, PCR products were visualised by agarose gel electrophoresis and submitted to SANGER sequencing. Phylogenetic analysis was performed with PhyML software that estimates maximum likelihood phylogenies from alignments of nucleotide sequences using the General Time Reversible model and 1,000 bootstrap replicates [15].

## 3. Rabies in Poland in 2021 and 2022

Poland has implemented ORV on its whole territory since 2002 and conducted an agreement with Ukraine to carry out vaccination in Ukraine along the Polish border (∼100 km wide) since 2012 [16]. Until 2020, the rabies epizootic situation in Poland was favourable, except for the rabies outbreak in Malopolska voivodeship in 2010 [17]. This positive trend was interrupted by a rabies outbreak in Mazowieckie voivodeship, which caused a drastic increase in rabies cases [18]. The last rabies cases in this voivodeship were indeed reported in 2004. Following this resurgence, 113 rabies cases in animals were reported both in wild and domestic animals. The highest number of rabies cases (*n* = 96; 84.95%) were registered in the red fox. Other species of animals, both wildlife and domestic, accounted for a small percentage of all diagnosed cases of the disease (*n* = 17; 15.05%). Since the detection of the first rabies case in the central part of Mazowieckie voivodeship, the number of diagnosed animal rabies cases increased, and the wave of rabies moved southwards, reaching the voivodeship of Świętokrzyskie in November 2021 (Figure 2). Considering the geographical distribution, the highest number of rabies cases were registered in the Mazowieckie voivodeship. Of all 113 diagnosed cases of rabies, 109 were from this area, which accounted for 96.46% of all registered rabies cases. Only two out of 113 rabies cases were recorded in Podkarpackie voivodeship at the beginning of 2021 (January and February).

In 2022, the evolution of the rabies situation showed a decrease in rabies incidence, with 36 rabies cases reported in terrestrial animals. A total of 3,836 animal samples were submitted under rabies surveillance of indicator animals, among which 2,212 (57.8%) samples originated from wildlife and 1,624 (42.2%) samples from domestic animals. Rabies cases were again most frequently diagnosed in foxes, with 32 cases representing 88.9% of the total number of rabies cases detected. The improvement of the epizootic situation of rabies was possible due to the continuation and extension of vaccination campaigns of wild animals and other rabies control measures implemented by the Polish authorities. Most of the diagnosed rabies cases were identified in the Mazowieckie voivodeship 31 (86.1%), while 3 and 2 rabies cases were recorded in Świętokrzyskie and Lubelskie voivodeship, respectively, as a result of rabies epizootic in Mazowieckie voivodeship. Up to March 2023, date of redaction of this manuscript, no case of rabies has been reported in animals in Poland.

## 4. Rabies in Hungary in 2022

Due to regular ORV campaigns on the whole territory, the number of rabies-positive terrestrial animals decreased from around 1,000 cases per year in the early 1990s to only sparse cases that did not exceed 10 per year since 2005, with the exception of 2013 and 2014 were 23 and 24 cases were detected, respectively [19]. The last classical rabies Hungarian cases were detected in March 2017 in a red fox and two goats [19]. Since this period, an ORV has been maintained in Hungary with bordering infected countries over at least 50 km wide to create an immune belt. In the Ukrainian territories bordering Hungary, ORV was performed over the autumn 2017–2021 period.

**Figure 3:**
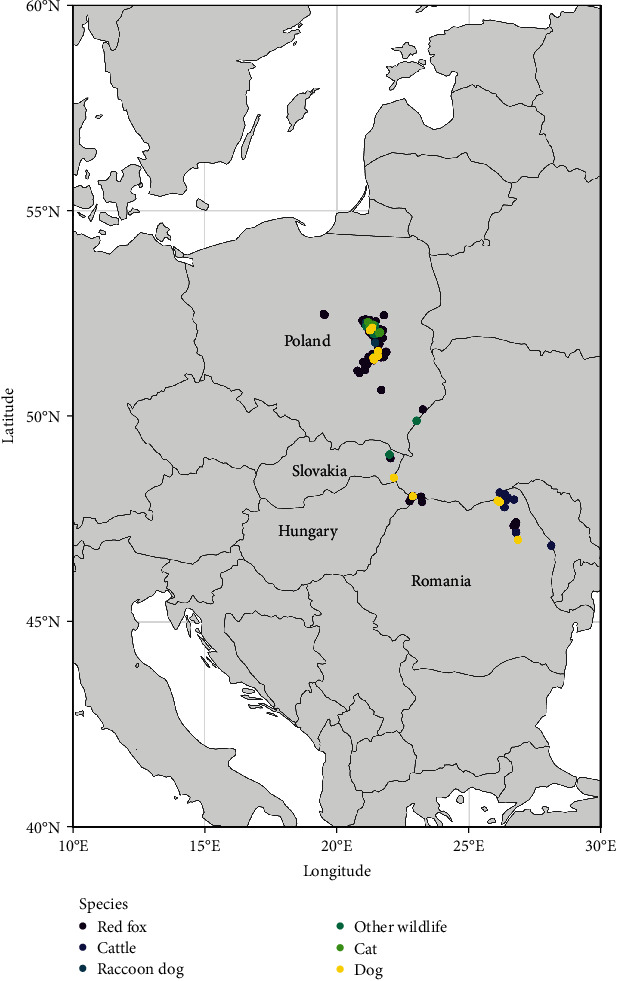
Distribution of species found infected by rabies in the EU during 2021–2022 (data source: Animal Disease Information System (ADIS) (europa.eu)).

In September 2022, a fox spotted in Botpalád, 5 km from the Ukrainian border, showing characteristic neurological symptoms, was shot by the hunters (Figure 3). The National Reference Laboratory of Hungary confirmed the presence of the classical rabies virus in the suspect animal. This first case detected since 5 years without rabies cases detected, was followed on 1 November by a dog case in Magosliget, about 5 km from the previous location, which also showed abnormal behaviour. On 10 November, another red fox was shot in the village of Uszka, only a few hundred metres away from the previous shooting site, and was also found to be rabies-positive. The last rabies-positive animal in 2022 was another fox, which was shot on 20 November 2022, a little further away from the previous areas (but still only 15 km from the Romanian–Ukrainian–Hungarian border), near Kisnamény. The latter two animals presented a similar profile, as they did not show any characteristic neurological signs. The examination of the teeth did not show the typical TTC biomarkers that appear after oral vaccine baits uptake. Both of them were young animals, so they may not had the opportunity to consume vaccine baits or, as they were shot during the autumn season when juveniles disperse, they were originated from a country without any ORV campaigns. On the date of redaction of this manuscript (March 2023), one case was detected in February in the same geographical district as previously. These cases were detected while passive surveillance data indicated a very slight decrease in surveillance pressure for the last years within the country (779 analyses in 2022, 894 in 2021, 872 in 2020, and 1077 in 2019). However, since the detection of cases, a surveillance increase, particularly linked to active surveillance, is locally observed.

## 5. Rabies in Slovakia in 2022

Thanks to ORV campaigns, rabies cases decreased dramatically from the 1990s to 2000s to reach 0 cases since 2007, with the exception of some cases detected during the period 2013–2015 (12 cases) (Figure 2). While ORV has still been continuously implemented creating wide immune belt bordering Ukraine, three positive cases of rabies were identified in 2022 (Figure 2).

In September 2022, a badger with unexpected behaviour was shot in the Jabloň village and confirmed positive by the Slovakian National Reference Laboratory (NRL). The village is located in the north-eastern part of Slovakia near the borders with Poland and Ukraine (Figure 3). After 7 years without rabies cases detected and constant surveillance pressure (from around 300 to 400 analysis was performed per year since 2016), this positive case led to increased rabies surveillance in the surrounding regions. This surveillance (passive and active) increased from 34 samples examined in 2021 to 77 samples in 2022 in district Humenne only. Various species of animals have been tested, including foxes, badgers, raccoon dogs, wild boars, dogs, and rodents. From these samples, one fox was confirmed positive for rabies (Figure 3). The fox was found dead on 29 December 2022, and the rabies virus was confirmed on 2 January 2023. The fox was found in the village Rovné nad Udavou, near the first outbreak of rabies. The area in which these cases were detected has been regularly covered by ORV for more than 20 years. In addition to wild animals, rabies virus was also confirmed in a dog in the Trenčín dog shelter (western part of Slovakia) in December 2022. A female of unknown origin with no microchip was captured together with three puppies near the village of Veľké Slemence. The village is located on the border with Ukraine, where is also a border crossing for pedestrians. Due to the full capacity of the nearby shelters, she and her puppies were taken to the dog shelter in Trenčín. During stay in the shelter, she began to show neurological symptoms and died. After laboratory examination, rabies virus was confirmed in the NRL. Subsequently, her three puppies and five other dogs from the shelter that were in contact with her were euthanized and sent for rabies testing. All these dogs were negative for rabies.

## 6. Rabies in Romania in 2021 and 2022

ORV programme was organised for the first time in 2011 in the western third of the country and has been implemented on the whole territory since 2012. Since then, ORV has been regularly completed, twice a year, as recommended (EC 2012). Unfortunately, they have experienced occasional interruptions, such as in 2012, 2013, in 2021 (spring and autumn) and in 2022 (spring), where no ORV campaign was achieved. Since 2018–2020, Romania has conducted an agreement with the Republic of Moldova and Ukraine to carry out ORV on their bordering with the Republic of Moldova territory along a 50 km wide band and a 100 km width belt on the Ukrainian territory, respectively.

The number of animal rabies cases detected in Romania reached its maximum, with 1138 cases in 2008. Five years after the start of the ORV, the number of cases has decreased significantly to less than 20 cases since 2016 and less than 5 cases since 2017.

In 2021, a total of five cases were detected. The first 2021 case was a fox collected in January close to Satu Mare, 20 km far from the Ukrainian border in the northern part of Romania. This case was followed, at the end of the year (over November–December) by four cattle cases detected in the eastern part of the country: three cattle cases in Iași county with two close to Dumbrava (20 km far from the Republic of Moldova) and one close to Gorban (at the border with the Republic of Moldova), and one cattle in Botosani county near Ibanesty (15 km far from Ukraine and 30 km far from Republic of Moldova). In 2022, the number of positive samples increased considerably compared to 2021 to reach a total of 28 cases (15 cattle, 6 dogs, and 7 foxes) (Figure 1). More than half of the cases were detected over the November–December period (*n* = 15, 60%), and all except one were detected in the eastern part of the country (Iasi, Botosani, and Suceava counties). Almost half of the species diagnosed positive were cattle (*n* = 12; 48%), which are not the primary source for rabies (Figure 3). Up to March 2023, date of redaction of this mancuscript, seven rabies cases have been notified in the country.

## 7. Phylogenetic Analysis of the 2021–2022 Cases

**Figure 4:**
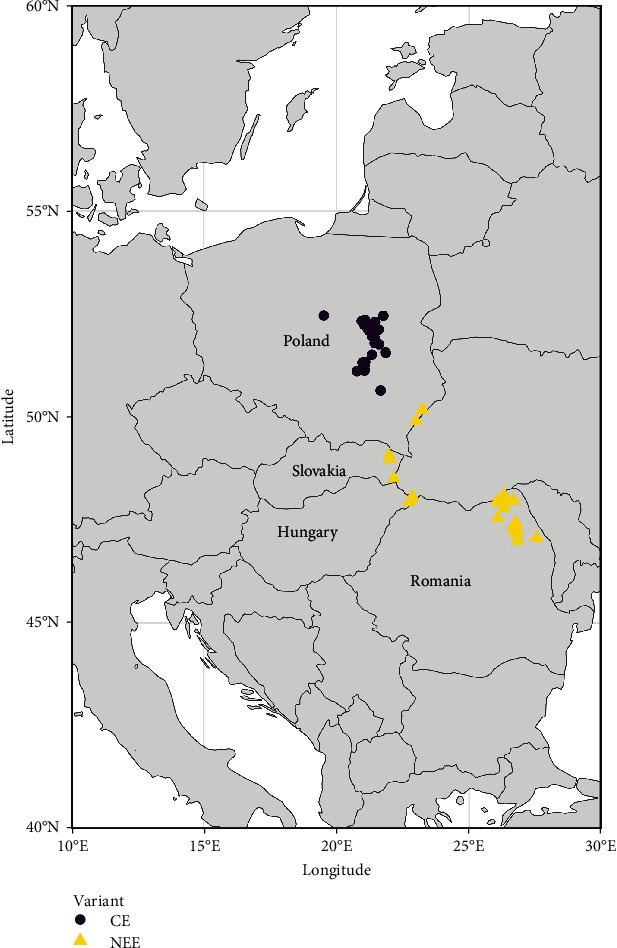
Geographical distribution of sylvatic rabies phylogroups isolated in the EU from 2021 to 2022.

**Figure 5:**
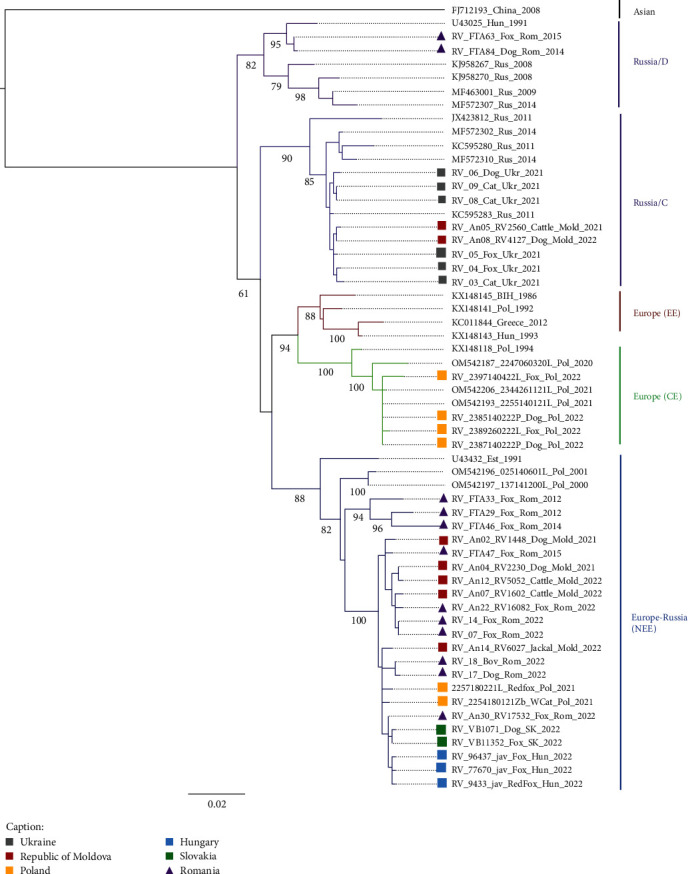
Phylogenetic relationships between the entire N gene sequences of 34 new cases detected during 2021–2022 and of 2 cases from 2014 to 2015 with RABV sequences representative of subgroups NEE, C, EE, CE, and D. Overall, 100% homological new sequences (*n* = 69) have been deleted from the analysis for tree visibility.

All rabies-positive samples detected in Slovakia (3/3 samples), Hungary (4/4 samples), and 30/33 samples (91%) identified in Romania over the period 2021–2022 were collected and included in the analysis, indicating a good representativeness of the study's samples within detected cases of the same period. For Poland, as a previous study analysed all 2021 cases and January 2022 cases, only a part of 2021 cases (11/113) were selected to be representatives of phylogroups previously detected, and 31/36 samples (86%) detected in 2022 were selected for the phylogenetic analysis. According to the results, all strains had a wild origin. Cases isolated in Poland in the Mazowieckie voivodeship and surroundings belonged to the Central Europe (CE) variant as previously described [18]. In contrast, all other cases detected in the 2021–2022 period in Hungary, Slovakia, Romania, and the eastern part of Poland were very distinct to the Mazowieckie outbreak and belonged to the North East Europe (NEE) phylogroup (Figures 4 and 5). The previously described NEE variant [20] historically extends from East to Central-Eastern Europe. The variant was indeed detected in western Russia, Ukraine, Baltic countries, Bulgaria, Hungary, Poland, Romania, and Slovakia [21–24]. This variant seems currently predominant in the EU as all isolated samples from 2021 to 2022, except from the Mazowieckie outbreak, belong to this phylogroup.

## 8. Conclusion

Central-Eastern Europe experienced in a 2 years period two distinctive sylvatic rabies re-emergences. These two emergences have been detected while no specific decrease in surveillance pressure has been observed in previously free from rabies countries. Thanks to the co-financed and regular implementation of ORV programmes in large areas, the EU has come over the period 2016–2019 close to the goal of eliminating rabies from its territory. However, the re-surgence of 2021 in Poland and at the end of 2022 in Hungary and Slovakia and the worsened situation in Romania highlighted the cruciality of sustainable long-term vaccination programmes to reach and maintain areas free of rabies, and the importance of maintaining coopertaive programmes with endemic bordering countries. Control measures of ORV implementation are also an important point to guarantee that no problems occur in ORV programmes. In countries with ORV programmes within the EU, for example, all oral vaccine batches used are previously analysed for bait titre verification. None of the titres analysed these last years have been found non-compliant.

One and a half years of ORV EU programmes interruption has dangerously brought Romania to a decreased sanitary condition in regards to rabies. The EU territory had not experienced such a situation since a decade. The rapid turnover of wildlife populations (wild red foxes have a life expectancy of 1–3 years) indeed does not allow the interruption of vaccination campaigns. Such interruption rapidly brings a non-negligible proportion of juvenile individuals in the reservoir population. These juvenile foxes are naïve to any exposure and, therefore, very susceptible to infection and are also likely for rabies dispersion due to their autumnal dispersal behaviour.

As in other parts of the world [25–27], the European rabies control system has also been challenged by the COVID-19 pandemic that has destabilised human societies and their organisation and probably induced perturbations in the animal rabies surveillance and its control system. Successive lockdowns of human populations most probably limited the wildlife disease surveillance in the field. Subsequent geopolitical conflicts affecting Ukraine have, among others, lead the flights of planes at the borders necessary to distribute oral vaccine baits almost impossible. Warfare, including shelling, air raids, and people movements, may have led to the frightening of animals, including those potentially incubating rabies, which left their habitats in search of safer ones, increasing the risk of spreading the rabies virus.

Indeed, the re-emergence of rabies in Hungary and Slovakia reminds the importance of maintaining a sufficiently wide immune belt along bordering endemic countries. Thus, previous cooperation ORV programmes have allowed maintaining at least 150 km wide immune belt, a width having demonstrated its effectiveness in previous years. Cooperation and mutual assistance between endemic rabies countries with bordering free (or almost free) countries is of paramount importance for reaching and maintaining rabies-free areas and for supporting endemic countries on the tortuous but achievable way to the elimination of rabies.

## Data Availability

Detail of samples included in the phylogenetic analysis is included in a table as supplementary material. Rabies case data notifications can be found in ADIS https://food.ec.europa.eu/animals/animal-diseases/animal-disease-information-system-adis_en.

## Supplementary Materials

Supplementary Materials The supplementary material lists and describes the sequences used for the phylogenetic analysis. It includes 29 references already recorded in the GenBank database and 105 new sequences included via this study. Table of sequences included in the phylogenetic analyses (29 references and 105 samples from this study).
