# Supplementary material for: Two Sylvatic Rabies Re-Emergences in Central-Eastern Europe over the 2021–2022 Period: An Unprecedented Situation in Recent Years
Source: Transbound Emerg Dis. 2023 Nov 10;2023:5589201. doi: 10.1155/2023/5589201 (PMC12016682; doi:10.1155/2023/5589201)
Supplement: Supplementary Materials — The supplementary material lists and describes the sequences used for the phylogenetic analysis. It includes 29 references already recorded in the GenBank database and 105 new sequences included via this study. Table of sequences included in the phylogenetic analyses (29 references and 105 samples from this study). [file 5589201.f1.docx]

Supplementary material: Table of sequences included in the phylogenetic analyses (29 references and 105 samples from this study)

| Country | Id Sample | Id sample (Phylo tree) | Year of isolation | Species | County | Phylogenetic clade | This study | GenBank accession number |
| --- | --- | --- | --- | --- | --- | --- | --- | --- |
| China | / | / | 2008 | Dog | / | Asian | no | FJ712193 |
| Russia | / | / | 2011 | Red fox | / | C | no | KC595280 |
| Russia | / | / | 2011 | Red fox | / | C | no | KC595283 |
| Russia | / | / | 2011 | Red fox | / | C | no | JX423812 |
| Russia | / | / | 2014 | Cat | / | C | no | MF572310 |
| Russia | / | / | 2014 | Red fox | / | C | no | MF572302 |
| Poland | / | / | 1994 | Raccoon dog | / | CE | no | KX148118 |
| Poland | / | / | 2020 | Red fox | / | CE | no | OM542187 |
| Bosnia and Herzegovina | / | / | 1986 | Wolf | / | EE | no | KX148145 |
| Greece | / | / | 2012 | Red fox | / | EE | no | KC011844 |
| Hungary | / | / | 1993 | Red fox | / | EE | no | KX148143 |
| Poland | / | / | 1992 | Red fox | / | EE | no | KX148141 |
| Estonia | / | / | 1991 | No data | / | NEE | no | U43432 |
| Poland | / | / | 2000 | Red fox | / | NEE | no | OM542197 |
| Poland | / | / | 2001 | Red fox | / | NEE | no | OM542196 |
| Hungary | / | / | 1991 | No data | / | Russia / D | no | UA3025 |
| Russia | / | / | 2008 | Red fox | / | Russia / D | no | KJ958267 |
| Russia | / | / | 2008 | Red fox | / | Russia / D | no | KJ958270 |
| Russia | / | / | 2009 | Red fox | / | Russia / D | no | MF463001 |
| Russia | / | / | 2014 | Raccoon dog | / | Russia / D | no | MF572307 |
| Poland | 2266140421L_POL |  | 2021 | Red fox | Mazovie | CE | no | OM542199 |
| Poland | 2276140521L_POL |  | 2021 | Red fox | Mazovie | CE | no | OM542200 |
| Poland | 2277140521J_POL |  | 2021 | Raccoon dog | Mazovie | CE | no | OM542201 |
| Poland | 2259140321L_POL |  | 2021 | Red fox | Mazovie | CE | no | OM542202 |
| Poland | 2283140621L_POL |  | 2021 | Red fox | Mazovie | CE | no | OM542203 |
| Poland | 2344261121L_POL |  | 2021 | Red fox | Swietokrzyskie | CE | no | OM542206 |
| Poland | 2255140121L_POL |  | 2021 | Red fox | Mazovie | CE | no | OM542193 |
| Poland | 2256140121L_POL |  | 2021 | Red fox | Mazovie | CE | no | OM542194 |
| Poland | 2258140221L_POL |  | 2021 | Red fox | Mazovie | CE | no | OM542195 |
| Moldova | 2560 | # RV_An05_RV2560_Cattle_Mold_2021 | 2021 | Cattle | Transnistria | C | yes | OQ656654 |
| Moldova | 5362 |  | 2022 | Cattle | Sîngerei | C | yes | OQ656659 |
| Moldova | 5434 |  | 2022 | Cattle | Sîngerei | C | yes | OQ656665 |
| Moldova | 4127 | # RV_An08_RV4127_Dog_Mold_2022 | 2022 | Dog | Sîngerei | C | yes | OQ656657 |
| Ukraine | # RV_03_Vinnytska_Cat_Ukr_2021 | # RV_03_Vinnytska_Cat_Ukr_2021 | 2021 | Cat | Vinnytska | C | yes | OQ627305 |
| Ukraine | # RV_04_Vinnytska_Fox_Ukr_2021 | # RV_04_Vinnytska_Fox_Ukr_2021 | 2021 | Red fox | Vinnytska | C | yes | OQ627306 |
| Ukraine | # RV_05_Vinnytska_Fox_Ukr_2021 | # RV_05_Vinnytska_Fox_Ukr_2021 | 2021 | Red fox | Vinnytska | C | yes | OQ627307 |
| Ukraine | # RV_06_Kirovohradska_Dog_Ukr_2021 | # RV_06_Kirovohradska_Dog_Ukr_2021 | 2021 | Dog | Kirovohradska | C | yes | OQ627308 |
| Ukraine | # RV_08_Rivnenska_Cat_Ukr_2021 | # RV_08_Rivnenska_Cat_Ukr_2021 | 2021 | Cat | Rivnenska | C | yes | OQ627309 |
| Ukraine | # RV_09_Rivnenska_Cat_Ukr_2021 | # RV_09_Rivnenska_Cat_Ukr_2021 | 2021 | Cat | Rivnenska | C | yes | OQ627310 |
| Poland | 2266140421L_POL |  | 2021 | Red fox | Mazovie | CE | no | OM542199 |
| Poland | 2276140521L_POL |  | 2021 | Red fox | Mazovie | CE | no | OM542200 |
| Poland | 2277140521J_POL |  | 2021 | Raccoon dog | Mazovie | CE | no | OM542201 |
| Poland | 2259140321L_POL |  | 2021 | Red fox | Mazovie | CE | no | OM542202 |
| Poland | 2283140621L_POL |  | 2021 | Red fox | Mazovie | CE | no | OM542203 |
| Poland | 2344261121L_POL |  | 2021 | Red fox | Swietokrzyskie | CE | no | OM542206 |
| Poland | 2255140121L_POL |  | 2021 | Red fox | Mazovie | CE | no | OM542193 |
| Poland | 2256140121L_POL |  | 2021 | Red fox | Mazovie | CE | no | OM542194 |
| Poland | 2258140221L_POL |  | 2021 | Red fox | Mazovie | CE | no | OM542195 |
| Poland | 2386060222L_POL |  | 2022 | Red fox | Swietokrzyskie | CE | yes | OQ693953 |
| Poland | 2388140222L_POL |  | 2022 | Red fox | Mazovie | CE | yes | OQ693955 |
| Poland | 2390060322L_POL |  | 2022 | Red fox | Lublin | CE | yes | OQ693957 |
| Poland | 2391140322L_POL |  | 2022 | Red fox | Mazovie | CE | yes | OQ693958 |
| Poland | 2393140222L_POL |  | 2022 | Red fox | Mazovie | CE | yes | OQ693959 |
| Poland | 2395140422L_POL |  | 2022 | Red fox | Mazovie | CE | yes | OQ693960 |
| Poland | 2398140422Br_POL |  | 2022 | Badger | Mazovie | CE | yes | OQ693962 |
| Poland | 2399260422L_POL |  | 2022 | Red fox | Swietokrzyskie | CE | yes | OQ693963 |
| Poland | 2400140522L_POL |  | 2022 | Red fox | Mazovie | CE | yes | OQ693964 |
| Poland | 2401140522L_POL |  | 2022 | Red fox | Mazovie | CE | yes | OQ693965 |
| Poland | 2402260522L_POL |  | 2022 | Red fox | Swietokrzyskie | CE | yes | OQ693966 |
| Poland | 2403140522L_POL |  | 2022 | Red fox | Mazovie | CE | yes | OQ693967 |
| Poland | 2404140522L_POL |  | 2022 | Red fox | Mazovie | CE | yes | OQ693968 |
| Poland | 2405140722L_POL |  | 2022 | Red fox | Mazovie | CE | yes | OQ693969 |
| Poland | 2406140722L_POL |  | 2022 | Red fox | Mazovie | CE | yes | OQ693970 |
| Poland | 2372140122L_POL |  | 2022 | Red fox | Mazovie | CE | yes | OQ693971 |
| Poland | 2373140122L_POL |  | 2022 | Red fox | Mazovie | CE | yes | OQ693972 |
| Poland | 2374140122L_POL |  | 2022 | Red fox | Mazovie | CE | yes | OQ693973 |
| Poland | 2375140122L_POL |  | 2022 | Red fox | Mazovie | CE | yes | OQ693974 |
| Poland | 2376140122L_POL |  | 2022 | Red fox | Mazovie | CE | yes | OQ693975 |
| Poland | 2377140122L_POL |  | 2022 | Red fox | Mazovie | CE | yes | OQ693976 |
| Poland | 2379140122L_POL |  | 2022 | Red fox | Mazovie | CE | yes | OQ693977 |
| Poland | 2380140122L_POL |  | 2022 | Red fox | Mazovie | CE | yes | OQ693978 |
| Poland | 2381140122L_POL |  | 2022 | Red fox | Mazovie | CE | yes | OQ693979 |
| Poland | 2382140122L_POL |  | 2022 | Red fox | Mazovie | CE | yes | OQ693980 |
| Poland | 2383140222L_POL |  | 2022 | Red fox | Mazovie | CE | yes | OQ693981 |
| Poland | 2384140222L_POL |  | 2022 | Red fox | Mazovie | CE | yes | OQ693982 |
| Poland | 2385140222P_POL | # RV_2385140222P_Dog_Pol_2022 | 2022 | Dog | Mazovie | CE | yes | OQ693952 |
| Poland | 2387140222P_POL | # RV_2387140222P_Dog_Pol_2022 | 2022 | Dog | Mazovie | CE | yes | OQ693954 |
| Poland | 2389260222L_POL | # RV_2389260222L_Fox_Pol_2022 | 2022 | Red fox | Swietokrzyskie | CE | yes | OQ693956 |
| Poland | 2397140422L_POL | # RV_2397140422L_Fox_Pol_2022 | 2022 | Red fox | Mazovie | CE | yes | OQ693961 |
| Poland | 2254180121Zb_POL | # RV_2254180121Zb_WCat_Pol_2021 | 2021 | Wild cat | Stubno | NEE | yes | OQ693988 |
| Poland | 2257180221L_POL |  | 2021 | Red fox | Basznia Dolna | NEE | yes | OQ693989 |
| Romania | FTA84 | # RV_FTA84_Dog_Rom_2014 | 2014 | Dog | Cerașu | D | yes | OQ656699 |
| Romania | FTA63 | # RV_FTA63_Fox_Rom_2015 | 2015 | Red fox | Maneciu | D | yes | OQ656698 |
| Hungary | 89368 |  | 2022 | Dog | Magosliget | NEE | yes | OQ693996 |
| Hungary | 94133 | # RV_9433_jav_RedFox_Hun_2022 | 2022 | Red fox | Uszka | NEE | yes | OQ693994 |
| Hungary | 96437 | # RV_96437_jav_Fox_Hun_2022 | 2022 | Red fox | Kisnameny | NEE | yes | OQ693997 |
| Hungary | 77670 | #RV_77670_jav_Fox_Hun_2022 | 2022 | Red fox | Botpalad | NEE | yes | OQ693995 |
| Moldova | 2561 |  | 2021 | Cattle | Transnistria | NEE | yes | OQ656652 |
| Moldova | 3287 |  | 2021 | Cattle | Transnistria | NEE | yes | OQ656655 |
| Moldova | 1448 | # RV_An02_RV1448_Dog_Mold_2021 | 2021 | Dog | Anenii Noi | NEE | yes | OQ656651 |
| Moldova | 2230 | # RV_An04_RV2230_Dog__Mold_2021 | 2021 | Dog | Transnistria | NEE | yes | OQ656653 |
| Moldova | 2940 |  | 2022 | Cat | Cantemir | NEE | yes | OQ656650 |
| Moldova | 3800 |  | 2022 | Red fox | Transnistria | NEE | yes | OQ656660 |
| Moldova | 4246 |  | 2022 | Cattle | Briceni | NEE | yes | OQ656662 |
| Moldova | 6176 |  | 2022 | Dog | Briceni | NEE | yes | OQ656664 |
| Moldova | 804 |  | 2022 | Cattle | Falesti | NEE | yes | OQ656658 |
| Moldova | 1602 | # RV_An07_RV1602_Cattle_Mold_2022 | 2022 | Cattle | Telenești | NEE | yes | OQ656656 |
| Moldova | 5052 | # RV_An12_RV5052_Cattle_Mold_2022 | 2022 | Cattle | Transnistria | NEE | yes | OQ656661 |
| Moldova | 6027 | # RV_An14_RV6027_Jackal_Mold_2022 | 2022 | Jackal | Briceni | NEE | yes | OQ656663 |
| Romania | FTA29 | # RV_FTA29_Fox_Rom_2012 | 2012 | Red fox | Șinca | NEE | yes | OQ656694 |
| Romania | FTA33 | # RV_FTA33_Fox_Rom_2012 | 2012 | Red fox | Bătarci | NEE | yes | OQ656695 |
| Romania | FTA46 | # RV_FTA46_Fox_Rom_2014 | 2014 | Red fox | Malaia | NEE | yes | OQ656696 |
| Romania | FTA47 | # RV_FTA47_Fox_Rom_2015 | 2015 | Red fox | Brâncoveni, | NEE | yes | OQ656697 |
| Romania | 13192 |  | 2020 | Red fox | Iasi | NEE | yes | OQ656666 |
| Romania | 13132 |  | 2020 | Cattle | Iasi | NEE | yes | OQ656667 |
| Romania | 16812 |  | 2020 | Cattle | Satu Mare | NEE | yes | OQ656668 |
| Romania | 17233 |  | 2021 | Cattle | Iasi | NEE | yes | OQ656669 |
| Romania | 17204 |  | 2021 | Cattle | Iasi | NEE | yes | OQ656670 |
| Romania | CA-IDSA-17242 |  | 2022 | Cattle | Iasi | NEE | yes | OQ656680 |
| Romania | CA-IDSA-17538 |  | 2022 | Dog | Neamt | NEE | yes | OQ656681 |
| Romania | CA-IDSA-17923 |  | 2022 | Cattle | Botosani | NEE | yes | OQ656682 |
| Romania | CA-IDSA-17792 |  | 2022 | Dog | Suceava | NEE | yes | OQ656683 |
| Romania | CA-IDSA-17350 |  | 2022 | Cattle | Iasi | NEE | yes | OQ656684 |
| Romania | CA-IDSA-17241 |  | 2022 | Cattle | Sirețel | NEE | yes | OQ656686 |
| Romania | CA-IDSA-17295 |  | 2022 | Dog | Suceava | NEE | yes | OQ656687 |
| Romania | CA-IDSA-17376 |  | 2022 | Dog | Suceava | NEE | yes | OQ656688 |
| Romania | CA-IDSA-17230 |  | 2022 | Dog | Suceava | NEE | yes | OQ656689 |
| Romania | CA-IDSA-17847 |  | 2022 | Cattle | Suceava | NEE | yes | OQ656690 |
| Romania | CA-IDSA-17324 |  | 2022 | Cattle | Sirețel | NEE | yes | OQ656691 |
| Romania | CA-IDSA-17351 |  | 2022 | Red fox | Iasi | NEE | yes | OQ656693 |
| Romania | 15340 |  | 2022 | Red fox | Iasi | NEE | yes | OQ656672 |
| Romania | 16362 |  | 2022 | Cattle | Botosani | NEE | yes | OQ656673 |
| Romania | 14992 |  | 2022 | Cattle | Iasi | NEE | yes | OQ656674 |
| Romania | 16921 |  | 2022 | Cattle | Iasi | NEE | yes | OQ656676 |
| Romania | 17092 |  | 2022 | Cattle | Iasi | NEE | yes | OQ656677 |
| Romania | 16698 | # RV_07_Iasi_Fox_Rom_2022 | 2022 | Red fox | Iasi | NEE | yes | OQ656671 |
| Romania | 16920 | # RV_14_Iasi_Fox_Rom_2022 | 2022 | Red fox | Iasi | NEE | yes | OQ656675 |
| Romania | 17139 | # RV_17_Suceava_Dog_Rom_2022 | 2022 | Dog | Suceava | NEE | yes | OQ656678 |
| Romania | 17148 | # RV_18_Botosani_Bov_Rom_2022 | 2022 | Cattle | Botosani | NEE | yes | OQ656679 |
| Romania | CA-IDSA-16082 | # RV_An22_RV16082_Fox_Rom_2022 | 2022 | Red fox | Berezlogi | NEE | yes | OQ656685 |
| Romania | CA-IDSA-17532 | # RV_An30_RV17532_Fox_Rom_2022 | 2022 | Red fox | Sirețel | NEE | yes | OQ656692 |
| Slovakia | VB747-2022_Badger_Jablon_SK |  | 2022 | Badger | Jablon | NEE | yes | OQ544454 |
| Slovakia | VB1071-2022_Dog_V.Slemence_SK | # RV_VB1071_V.Slemence_SK_Dog_SK_2022 | 2022 | Dog | Velke Slemence | NEE | yes | OQ544455 |
| Slovakia | VB1135-2022_Fox_Rovne_SK | # RV_VB1135_Rovne_SK_Fox_SK_2022 | 2023 | Red fox | Rovne nad Udavou | NEE | yes | OQ544456 |
